# The epidemiological pattern of seasonal influenza in four sentinel sites in Iraq

**DOI:** 10.1111/irv.13134

**Published:** 2023-04-23

**Authors:** Ahmed Hasan Radhi, Ziyad Hazim Ibrahim, Riyadh Alhilifi

**Affiliations:** ^1^ CDC, MoH Baghdad Iraq; ^2^ Iraqi Ministry of Health Baghdad Iraq

**Keywords:** influenza, seasonal influenza, sentinel sites

## Abstract

**Introduction:**

Influenza is an acute viral infection with significant morbidity and mortality. It occurs annually each winter, which is called seasonal influenza, and is preventable through safe vaccine.

**Aim:**

The aim of this work is to know the epidemiological pattern of patients with seasonal influenza in Iraqi sentinel sites.

**Methods:**

A cross‐sectional study was carried out on records of patients who attended four sentinel sites and registered to have influenza‐like illness (ILI) or severe acute respiratory infection (SARI), and laboratory investigated.

**Results:**

The total number of cases was 1124; 36.2% of them aged 19–39 years; 53.9% were female; 74.9% lived in urban areas; 64.3% diagnosed as ILI; and 35.7% as SARI; 15.9% had diabetes, 12.7% had heart disease, 4.8% had asthma, 3% had a chronic lung disease, and 2% had hematological disease; 94.6% did not get influenza vaccine. About COVID‐19 vaccine, 69.4% were not vaccinated, 3.5% got only one dose, and 27.1% completed two doses. Only the SARI cases needed admission; among them, 95.7% were cured. 6.5% were diagnosed with influenza‐A virus, 26.1% had COVID‐19, and 67.5% were negative. Among those with influenza, 97.3% had H3N2 subtype and 2.7% had H1N1 pdm09.

**Conclusions:**

The percentage of influenza virus in Iraq is relatively small. The age, classification of case (ILI or SARI), having diabetes, heart disease, or immunological disease, and taking COVID‐19 vaccine have a significant association with influenza.

**Recommendations:**

It is needed for similar sentinel sites in other health directorates and for rising health education about seasonal influenza and its vaccine.

## 1 INTRODUCTION

Influenza is an acute viral respiratory infection that causes significant morbidity and mortality worldwide. Three types of influenza virus could cause disease in humans: A, B, and C. Influenza A is the most type responsible for causing pandemics because of its high susceptibility to antigenic variation.^1^ Influenza is a common virus whose ability to change its genetic makeup allows for a disease of pandemic proportion.^2^ It is a highly contagious and deadly virus, killing nearly half a million people yearly worldwide. The classic symptoms of the disease are fever, fatigue, cough, and body aches. In the outpatient setting, diagnosis can be made by clinical presentation with optional confirmatory diagnostic testing.^3^ The outbreak of influenza virus that usually occurs annually each winter is called seasonal influenza.^4^ In a majority of cases, seasonal influenza is preventable through safe and readily available vaccinations.^5^ The World Health Organization (WHO) estimated that seasonal influenza epidemics could cause three to five million severe cases annually and 290 000 to 650 000 deaths globally. Infection with seasonal influenza is mainly caused by influenza virus type A and type B.^6^, ^7^

Pregnant women, young children, the elderly, and persons with chronic illnesses are at high risk for severe illness and death associated with influenza viral infection. Seasonal influenza vaccination is the most effective way to prevent the infection and its complications.^8^ The vaccines are almost available but must be regularly updated.^6^ Understanding geographical and temporal patterns of seasonal influenza can help strengthen influenza surveillance to early detect epidemics and inform influenza prevention and control programs.^9^

Sentinel surveillance is defined as the monitoring of rate of occurrence of specific diseases/conditions through a voluntary network of doctors, laboratories, and public health departments with a view to assess the stability or change in health levels of a population.^10^ Data collected in a well‐designed sentinel system can be used to signal trends, identify outbreaks, and monitor disease burden, providing a rapid and economical alternative to other surveillance methods. Sentinel systems involve a network of reporting sites, typically doctors, laboratories, and public health departments.^11^

Knowledge of the epidemiological pattern among patients with seasonal influenza in Iraqi sentinel surveillance sites could be helpful in (1) early detection and rapid response to influenza outbreaks, (2) monitoring the trends of the disease, (3) monitoring the circulating influenza virus, (4) contributing to vaccine strain selection, (5) measuring the disease burden in Iraq, (6) establishing the determinants and risk factors for the diseases, (7) supporting the evidence‐based Iraqi health policy making, and (8) assessing the effect of Iraqi public health interventions regarding the influenza infection.

## 2 METHODOLOGY

It is a cross‐sectional study design. The four sites of Iraqi sentinel surveillance for seasonal influenza were selected to be the study settings; two are located in Baghdad; the capital (which are Al‐Nu'man Teaching Hospital in Rusafa “the eastern side” and Imamain Kadhimain Medical City in Karkh “the western side”), one in Erbil; in the north (Erbil Teaching Hospital), and one in Basra; in the south (Basra Teaching Hospital). The study populations in this work are all Iraqi patients, and all were recorded to have influenza‐like illness (ILI) or severe acute respiratory infection (SARI). The sampling was carried out for those cases who did match the inclusion criteria of both categories according to the WHO definition, which were registered in the four mentioned surveillance sites, from Nov. 2021, which is the date of establishing of those sites, till May 2022. There are two sentinel sites responsible for registration of cases with ILI, which are Al‐N'uman and Erbil Teaching Hospitals, while the other two sites are responsible for registration of cases with SARI “according to the WHO definition.” Regarding the tools of the study, this work involved the epidemiological characteristics of the participants according to their filled out special standard case‐investigation forms, which were registered in the mentioned four surveillance hospitals. These characteristics include the following variables: Age, sex, status of pregnancy, address, present and past medical history, classification of case (ILI or SARI), presence of some medical risk factors, status of immunization against influenza and COVID‐19, patient admission status and severity, final fate, type of sample, and type of virus and its strain after laboratory analysis at the Central Public Health Laboratory. The gathered data were coded for each participant and analyzed by SPSS 20 program, and then the data were summarized, analyzed, and presented as tables and charts. Chi‐square tests were done for comparison between the variables, and *p* value of <0.5 was considered in the determination of presence of any significant statistical association between those variables.

## 3 RESULTS

The total number of cases that were registered by the four sentinel surveillance sites for seasonal influenza in Iraq since their establishment in Nov. 2021 till May 2022 was 1124. The distribution of those cases regarding some personal and clinical variables is illustrated in Table 1, while their distribution regarding the presence of health problems is shown in Table 2.

**TABLE 1:** Frequency distribution and percentage of cases registered by sentinel surveillance sites for seasonal influenza in Iraq, *N* = 1124.

Variable		Frequency	Percentage
Site of surveillance	Baghdad/Rusafa	433	38.5
	Baghdad/Karkh	315	28
	Erbil	290	25.8
	Basra	86	7.7
Age classification	1–12	35	3.1
	13–18	79	7
	19–39	407	36.2
	40–59	383	34.1
	60 and more	220	19.6
Gender	Male	518	46.1
	Female	606	53.9
Pregnancy	Pregnant	7	1.1^a^
	Not pregnant	599	98.9^a^
Type of living area	Urban	842	74.9
	Rural	282	25.1
Classification of the case	ILI	723	64.3
	SARI	401	35.7
Presence of cough		1039	92.4
Presence of fever		1026	91.3
Sampling from upper respiratory tract		1124	100
Contact with birds		1	0.1
Last contact with similar case		1	0.1

^a^ Among the female patients.

**TABLE 2:** Frequency distribution and percentage of cases regarding the presence of health problems, *N* = 1124.

Variable	Frequency	Percentage
Hematological disease	22	2
Diabetes	179	15.9
Chronic lung disease	34	3
Asthma	54	4.8
Heart disease	143	12.7
Immunological disease	3	0.3
Chronic muscular disease	2	0.2
Chronic renal disease	7	0.6
Chronic neurological disease	6	0.5

**FIGURE 1:**
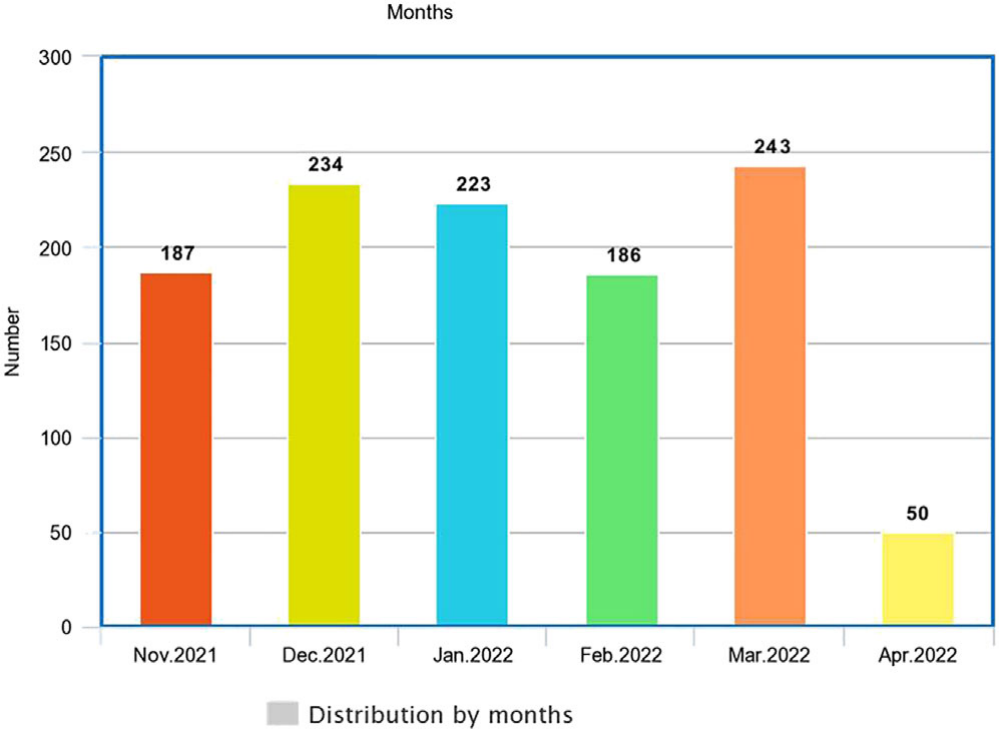
The time distribution of all cases in the four Iraqi sentinel sites for seasonal influenza concerning the month of attendance, *N* = 1124.

The most common cases were distributed in December and March respectively as shown in Figure 1.

**FIGURE 2:**
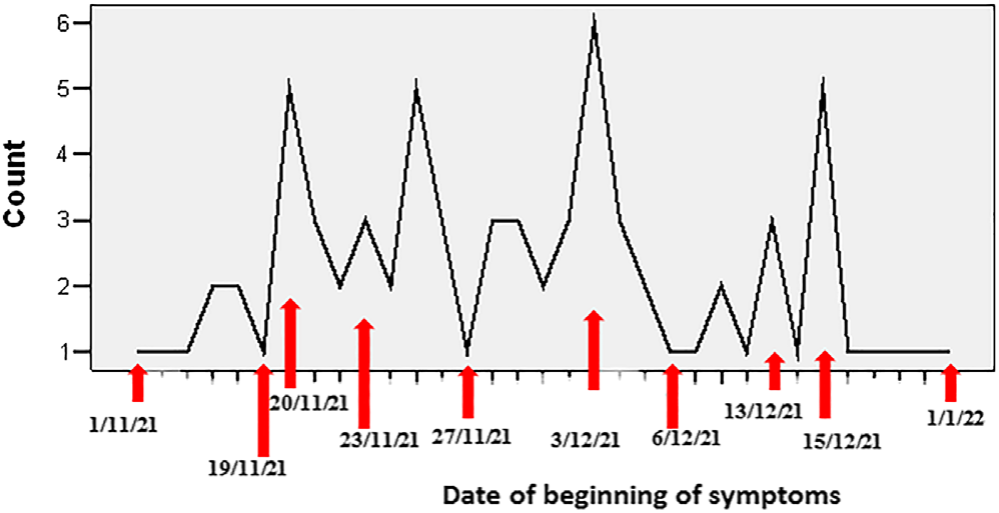
The date distribution of cases with influenza A virus who attended Iraqi sentinel sites, *n* = 73.

The total number of cases of influenza A virus who attended Iraqi sentinel sites was 73; most cases were distributed at the end of November and the first half of December as shown in Figure 2.

About 95% of all SARI cases were cured, and only 3.74% died; regarding the oxygen needed, about 16% were received as shown in Table 3.

**TABLE 3:** Frequency distribution and percentage of cases registered by the sentinel sites regarding status of hospitalization, *N* = 1124.

Variable		Frequency	Percentage
Admission status	Yes	401	35.7
	No	723	64.3
Needed for	Endotracheal intubation	1	0.25^a^
	Oxygen mask	65	16.21^a^
	C‐pap	4	1^a^
Final fate	Cured	384	95.7^a^
	Refereed	2	0.5^a^
	Dead	15	3.74^a^

^a^ Among admitted patients.

The distribution of personal, demographic, and clinical variables among patients who had influenza virus and those who did not have influenza virus and their association are shown in Table 4.

**TABLE 4:** The distribution of personal, demographic, and clinical variables among cases confirmed to have influenza virus and those who had no, and their association, *N* = 1124.

Variable		Have influenza virus	Have no influenza virus	*p* value
Age group	Less than 40	55	466	0.00001*
	40 and more	18	585	
Gender	Male	35	483	0.41
	Female	38	568	
Status of pregnancy	Pregnant	0	7	0.49
	Not pregnant	38	561	
Living area	Urban	60	782	0.08
	Rural	13	269	
Case classification	ILI	59	664	0.001*
	SARI	14	387	
Hematological disease	Yes	2	20	0.42
	No	71	1031	
Had diabetes	Yes	4	175	0.005*
	No	69	876	
Chronic lung disease	Yes	1	33	0.33
	No	72	1018	
Had asthma	Yes	4	50	0.47
	No	69	1001	
Heart disease	Yes	1	142	0.001*
	No	72	909	
Immunological disease	Yes	2	1	0.01*
	No	71	1050	
Muscular disease	Yes	0	2	0.87
	No	73	1049	
Chronic renal disease	Yes	0	7	0.62
	No	73	1044	
Neurological disease	Yes	0	6	0.66
	No	73	1045	
Contact with birds	Yes	0	1	0.93
	No	73	1050	
Contact with similar case	Yes	0	1	0.93
	No	73	1050	
Get influenza vaccine	Yes	6	55	0.19
	No	67	996	
Get COVID‐19 vaccine	1 dose only	10	29	0.001*
	2 doses	17	288	
	No	46	734	

* Statistically significant.

## 4 DISCUSSION

The total number of patients that were registered in those sites was 1124. Among those patients, about two thirds (64.3%) were enrolled as ILI and about one third (35.7%) were enrolled as SARI, which required admission to a hospital. More than two thirds of all cases (66.5%) were registered in Baghdad, which could reflect the high population density in the Iraqi capital, in which more than 7.5 million are living.^12^, ^13^ The largest age groups among the Iraqi patients who attended those sentinel sites are 19–39 years (36.2%) followed by 40–59 years (34.1%), on the contrary of the findings of another study done in Spain in which the most age group was less than 5 years old.^14^ Most of the registered patients in those Iraqi sentinel sites for seasonal influenza (74.9%) are living in urban areas; this could be related to their high awareness about respiratory symptoms during COVID‐19 pandemic, which lead the people to attend hospitals when they had fever and/or cough, or to the relative proximity of the sentinel sites to their house or residency. Regarding the clinical characteristics, vast majority of the registered cases had cough and fever (92.4% and 91.3% respectively); this is because of the case definition of ILI and SARI classification in which both categories have cough and fever in their definition. Nearly all cases that were registered in the four Iraqi sentinel sites had no contact with birds or with a similar case; this could reflect the presence of contact with unrecognized or denied cases. Concerning presence of comorbidities, the most accompanying health problems among the attended patients were diabetes mellitus and heart diseases (15.9% and 12.7%, respectively). Those diseases are considered groups at risk of influenza viral complications.^1^, ^15^ Regarding the date distribution of the registered cases, it is clear that the most months of high attendance of patients to those sites are March 2022 and December 2021 (21.6% and 19.8% consequently). Regarding the immunization status, the vast majority of cases (94.6%) did not take influenza vaccine, most of them (69.4%) did not take any dose of COVID‐19 vaccine, and only about quarter of them (27.1%) did complete the two doses of COVID‐19 vaccine. The percentage of patients in the current study who had no immunization against influenza virus is more than that founded by another study carried out in the United States, which showed that the percent of patients with SARI who did not get influenza vaccine was 38.9%.^16^ Concerning the status of hospitalization, only the SARI cases (35.7%) needed admission, which is compatible with the WHO definition. Among the admitted patients, some cases (16.2%) needed oxygen, and very little numbers required C‐pap or endotracheal intubation (1% and 0.25%, respectively). Vast majority of the admitted cases (95.7%) were discharged from the hospitals with cure, and some (3.7%) had died unfortunately. This goes with the fact that most of influenza infections considered as mild illnesses and the serious complications occur in a minority of cases.^6^ All the samples that were sent for laboratory diagnosis among the patients who attended Iraqi sentinel sites for seasonal influenza were taken from the upper respiratory tract. After sending the samples for a final confirmatory laboratory diagnosis, about quarter (26%) of the registered cases who attended those sentinel sites had COVID‐19, and a small percentage (6.5%) of them had infection with influenza virus type‐A. This small percentage goes with the fact that the activity of influenza virus declined during COVID‐19 pandemic substantially, which could be due to the preventive measures taken against COVID‐19 that may reduce other respiratory diseases from spreading.^17^ No one among the patients who attended those sentinel sites appeared to have the other types of influenza virus (type‐B or type‐C). It is manifested in the current study that the two common strain subtypes of influenza A virus in Iraq among those who were confirmed to have infection with influenza virus are H3N2 (97.3%) and H1N1 pdm09 (2.7%), while globally for 2018 to 2019 season “based on public health laboratory specimens,” the predominant influenza A subtype was H1N1 pdm09 (56.6% of positive specimens), followed by the H3N2 subtype (43.4%).^1^ Concerning the date distribution of beginning of symptoms for patients that were diagnosed to have influenza viral infection among cases registered in Iraqi sentinel sites from November 2021 till May 2022, it is revealed in this study that the case symptoms increased on November 20, November 25, December 3, and December 15, 2021; this fluctuation could be due to the environmental and weather circumstances in Iraq or the period of communicability of influenza virus, which might reach up to 7 days. The characteristics that appeared to have a significant statistical association with influenza viral infection are as follows: The age, classification of case (whether ILI or SARI), diabetes mellitus, heart disease, immunological disease, and the immunization status of COVID‐19 vaccine. It has appeared in this study that patients with higher risk of getting the influenza viral infection among those who attended Iraqi influenza surveillance sentinel sites from November 2021 till May 2022 are those aged less than 40 years, the cases that diagnosed as ILI, those who had diabetes mellitus, a heart disease, or an immunological disease, and those who never get the immunization with COVID‐19 vaccine. Another Brazilian study showed that the influenza viral infection had a significant association with age (as shown in our findings) and with cases that were diagnosed as SARI (on the contrary of the current study).^18^

## 5 CONCLUSION

1. Regarding the sociodemographic characteristics, Baghdad sentinel sites registered the most number of cases, the largest age groups among patients was 19–39 and 40–59 years, and most of them were living in urban areas.
2. Clinically, the percentage of ILI cases is more than that of SARI cases; the vast majority of patients presented with cough and fever; the diabetes mellitus and heart diseases are the most registered comorbidities among them.
3. Concerning the final confirmatory diagnosis for patients who attended Iraqi sentinel sites, only 6.5% of them had infection with influenza A virus. The strain subtypes of influenza A virus in Iraq among them were both H3N2 and H1N1 pdm09.
4. The age, classification of case (ILI or SARI), having diabetes, heart disease, or immunological disease, and taking COVID‐19 vaccine are the variables that appeared to have a significant association with influenza viral infection.

## 6 RECOMMENDATIONS

1. Establishment of other similar sentinel sites in other provinces is required in order to get an early detection of a large number of cases, to get more accurate data, and to cover more Iraqi governorates.
2. Focus on those who are aged 20–60 years, and on those with diabetes, heart disease, or immunological disease for enhancement of their health education about seasonal influenza and for encouraging them to take the influenza vaccine annually.
3. It is needed to increase the coverage of COVID‐19 vaccine in Iraq and to motivate on taking the two required doses.

## AUTHOR CONTRIBUTIONS

**Ahmed Hasan Radhi**: Project administration (equal); writing—review and editing. **Ziyad Ibrahim**: Data curation (equal); formal analysis (equal); methodology (equal); software (equal). **Riyadh Alhilfi**: Conceptualization (equal); supervision; visualization (equal).

## CONFLICT OF INTEREST STATEMENT

The authors declare no conflict of interest.

### PEER REVIEW

The peer review history for this article is available at https://www.webofscience.com/api/gateway/wos/peer-review/10.1111/irv.13134.

## Data Availability

The data are available from the corresponding author upon reasonable request.
